# Prevalence of *Plasmodium* spp. in *Anopheles* mosquitoes in Thailand: a systematic review and meta-analysis

**DOI:** 10.1186/s13071-022-05397-2

**Published:** 2022-08-06

**Authors:** Chutipong Sukkanon, Frederick Ramirez Masangkay, Wanida Mala, Kwuntida Uthaisar Kotepui, Polrat Wilairatana, Theeraphap Chareonviriyaphap, Manas Kotepui

**Affiliations:** 1 Medical Technology, School of Allied Health Sciences, Walailak University, Tha Sala, Nakhon Si Thammarat, Thailand; 2 Department of Medical Technology, Faculty of Pharmacy, University of Santo Tomas, Manila, Philippines; 3 Department of Clinical Tropical Medicine, Faculty of Tropical Medicine, Mahidol University, Bangkok, Thailand; 4 Department of Entomology, Faculty of Agriculture, Kasetsart University, Bangkok, Thailand; 5 Royal Society of Thailand, Sanam Suea Pa, Dusit, Bangkok, Thailand

**Keywords:** *Plasmodium*, *Anopheles*, Thailand, Meta-analysis

## Abstract

**Background:**

The entomological inoculation rate (EIR) is one of the key indices used to evaluate malaria transmission and vector control interventions. One of the components of the EIR is the sporozoite rate in *Anopheles* vectors. A systematic review and meta-analysis was performed to identify the prevalence of *Plasmodium* spp. in field-collected *Anopheles* species across Thailand.

**Methods:**

This systematic review was registered under the PROSPERO number CRD42021297255. Studies that focused on the identification of *Plasmodium* spp. in *Anopheles* mosquitoes were identified from the electronic databases PubMed, Web of Science, and Scopus. The quality of the identified studies was determined using the Strengthening the Reporting of Observational Studies in Epidemiology approach. The proportion of *Anopheles* mosquitoes collected, *Anopheles* vectors for *Plasmodium* species, and specificity of *Anopheles* vectors for *Plasmodium* species were analyzed. The pooled prevalence of *Plasmodium* species among the primary vectors (*Anopheles dirus*, *Anopheles minimus*, and *Anopheles maculatus*) was estimated using the random-effects model.

**Results:**

Of the 1113 studies identified, 31 were included in the syntheses. Of the 100,910 *Anopheles* mosquitoes identified for species and sibling species, *An. minimus* (40.16%), *An. maculatus* (16.59%), and *Anopheles epiroticus* (9.18%) were the most prevalent *Anopheles* species. Of the 123,286 *Anopheles* mosquitoes identified, 566 (0.46%) were positive for *Plasmodium* species. The highest proportions of *Plasmodium* species were identified in *Anopheles hodgkini* (2/6, 33.3%), *Anopheles nigerrimus* (2/24, 8.33%), *Anopheles balabacensis* (4/84, 4.76%), *An. dirus* (114/4956, 2.3%), *Anopheles annularis* (16/852, 1.88%), *Anopheles kochi* (8/519, 1.54%), *Anopheles vagus* (3/215, 1.4%), and *Anopheles baimaii* (1/86, 1.16%). The pooled prevalence of *Plasmodium* species identified in the main *Anopheles* vectors was 0.4% of that of *Plasmodium* species identified in *An. dirus* was 2.1%, that of *Plasmodium* species identified in *An. minimus* was 0.4%, and that of *Plasmodium* species identified in *An. maculatus* was 0.4%.

**Conclusions:**

We found a low prevalence of *Plasmodium* infection in *Anopheles* mosquitoes across Thailand. Therefore, the use of EIR to determine the impact of vector control intervention on malaria parasite transmission and elimination in Thailand must be undertaken with caution, as a large number of *Anopheles* specimens may be required.

**Graphical Abstract:**

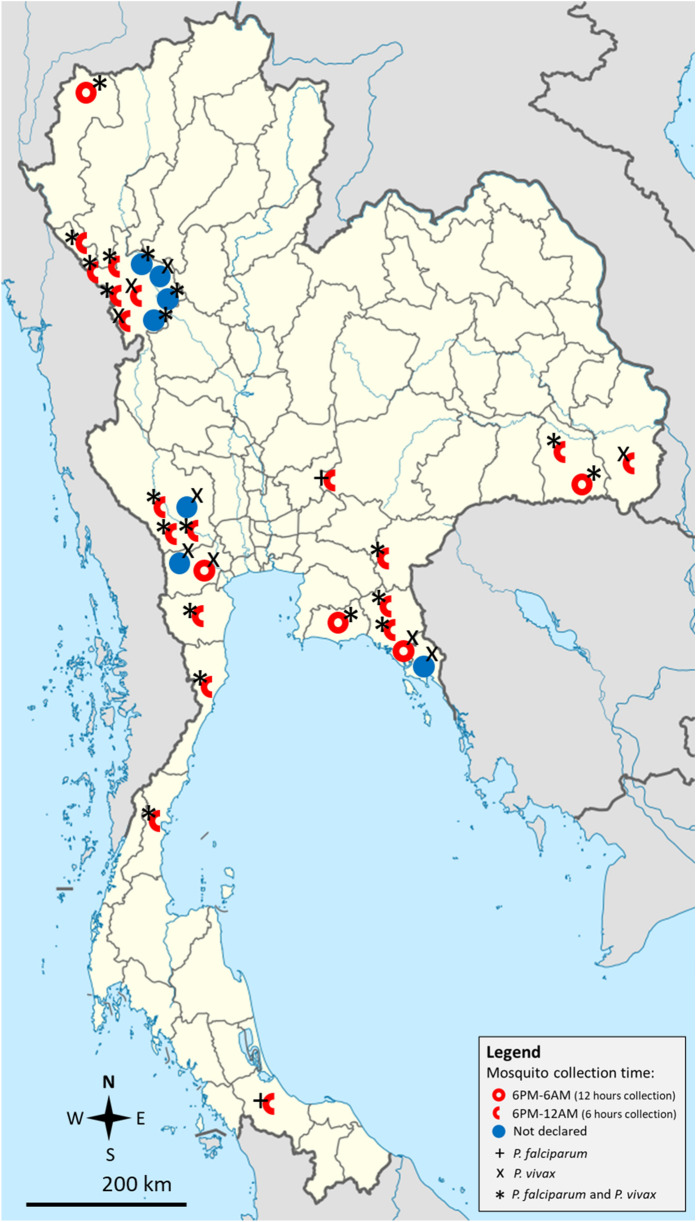

**Supplementary Information:**

The online version contains supplementary material available at 10.1186/s13071-022-05397-2.

## Background

Malaria is one of the major causes of morbidity and mortality in the Greater Mekong Subregion countries, including Thailand [1, 2]. Thailand is located at the center of the Indochinese Peninsula in Southeast Asia. It shares a border with Myanmar, Laos, Cambodia, and Malaysia, leading to a constant migration of foreign workers and refugees across the borders [3]. The presence of efficient vectors and long rainy season in Thailand indicates that there are several areas that can serve as mosquito habitats and that mosquito-borne diseases present a life-threatening public health challenge from both indigenous and imported cases [4]. Despite ongoing efforts to combat malaria, approximately 4473 confirmed cases were reported in Thailand in 2020. *Plasmodium vivax* is the dominant cause of infection (4098 cases, 92%), and the number of cases involving *Plasmodium falciparum* is much lower (256 cases, 5.7%) but still significant [5]. In Thailand, malaria cases are reported to have a high prevalence in vulnerable forest and forest fringes along rural stretches of border areas [3, 4]. Tak Province, which is a neighbor to Myanmar, had one of the highest incidences of malaria in Thailand in 2020 (1241 cases), followed by Yala Province in peninsular Thailand, on the border with Malaysia (1075 cases), and Kanchanaburi Province, another neighbor of Myanmar, with 539 confirmed cases [5]. Military personnel, forest workers, refugees, and local migrants are the individuals at the highest risk [3]. Although the malaria cases in Thailand in 2020 decreased by 24% compared to those in 2019 (5859 cases) [5], considerable effort is needed to achieve malaria elimination by 2024 [6]. In addition to techniques for the diagnosis of malaria and effective therapy, mosquito control is one of the most effective interventions against malaria. Distribution of insecticide-treated nets (ITNs) and indoor residual spraying (IRS) are currently used [5, 7]. With the implementation of the National Malaria Elimination Strategy 2017–2026, ITNs have started being distributed at a ratio of one ITN per two people, with the aim of at least 90% coverage in each transmission focus. Retreatment of the net is performed regularly every 6–12 months. However, if the retreatment process is inaccessible, long-lasting insecticidal nets (LLINs) are distributed. IRS is implemented in cases where both ITNs and LLINs cannot be allocated [7]. In 2020, over 102,150 LLINs were distributed with an average of 75% coverage across Thailand [5]. Unfortunately, these methods tackle only indoor- and late-biting vectors, and indoor-resting *Anopheles* mosquitoes are not suitable for reducing outdoor transmission. Thus, additional approaches are needed to protect people from outdoor- and early-biting vectors [8].

The malaria parasite infects humans through the bite of infected female *Anopheles* mosquitoes. Of the 540 *Anopheles* species described, 79 species are found in Thailand [9]. These species are normally classified into species complexes to which they are closely related and phenotypically indistinguishable [10]. However, due to their heterogeneity in ecology, bionomic pattern, and vectorial capacity across their distribution, different members of the species complex present different epidemiological roles in malaria transmission [9, 10]. Therefore, accurate species identification is important for evaluating transmission dynamics and applying the most appropriate vector control interventions [10]. In Thailand, seven species have been implicated as primary malaria vectors, in the Minimus and Dirus complexes and the Maculatus Group [11–14]. These species include *Anopheles dirus* Peyton & Harrison and *Anopheles baimaii* Sallum & Peyton of the Dirus Complex [15, 16]; *Anopheles minimus* Theobald of the Minimus Complex [15, 17]; *Anopheles aconitus* Dönitz of the Aconitus Subgroup (Funestus Group) [16, 18]; and *Anopheles maculatus* Theobald, *Anopheles pseudowillmori* Theobald, and *Anopheles sawadwongporni* Rattanarithikul & Green of the Maculatus Group [15, 19, 20]. Additionally, *Anopheles campestris* Reid, *Anopheles barbirostris* van der Wulp (Barbirostris Group) [21, 22], and *Anopheles epiroticus* Linton & Harbach (Sundaicus Complex) [23] have also been identified as potential malaria vectors in Thailand. Using serological or molecular assays, the rates of natural *Plasmodium* infection in the Dirus Complex, Minimus Complex, Maculatus Group, Barbirostris Group, and Sundaicus Complex were 0.8–6.4%, 0.09–5%, 0.1–3.1%, 0.42–1.9%, and 0.97%, respectively [10]. This information is crucial in determining the vector capacity of *Anopheles* species [24], as well as in planning vector control strategies.

To assess the transmission dynamics as well as the effectiveness of the vector control methods, it has been suggested that the entomological inoculation rate (EIR) should be evaluated annually [25]. The EIR measures the frequency of infectious bites by an *Anopheles* mosquito per person over time, combining the human biting rate and the sporozoite rate (SR) [26]. However, not all infectious bites result in blood-stage malaria in human hosts because *Plasmodium* parasites possibly experience significant bottlenecks during their sporogony cycle in mosquitoes [27]. The SR remains one of the important entomological indicators not only in assessing EIR but also in identifying *Anopheles* vectors that contribute to *Plasmodium* transmission in endemic settings [28]. Indicators other than EIR could also influence the transmission dynamics. As EIR can be estimated from the human biting rate, it has been demonstrated that very high vector density and high SR could sustain malaria transmission over a large part of the year [29]. In view of the evaluation of pre-oocyst formation blocking interventions, (e.g., gametocytocidal drugs), a previous study suggested that the oocyst formation rate is another highly reliable entomological indicator of mosquito infectiveness [30]. Although research into naturally infected *Anopheles* mosquito has been conducted for decades, there is a need for a comprehensive systematic review and meta-analysis focusing on the prevalence of malaria parasites in field-collected *Anopheles* species across Thailand. We herein mainly focused on the combined prevalence of both sporozoite and oocyst infection rates, hereby termed “*Plasmodium* infection.” The information collected and synthesized in this study improves our understanding of the local transmission dynamics of malaria vector species, particularly primary vectors, and may offer useful data for the evaluation of vector control interventions and malaria transmission.

## Methods

### Protocol

The systematic review followed the Preferred Reporting Items for Systematic Reviews and Meta-Analyses guidelines [31]. The systematic review was registered at PROSPERO with the number CRD42021297255.

### Literature search

Relevant studies were searched from three research databases, namely, PubMed, Web of Science, and Scopus, from inception to March 30, 2021. The reference lists of the included studies were also examined to ensure that relevant studies were not missed. We combined relevant search terms with Boolean operators, and the terms “(malaria OR *Plasmodium*) and (anopheles OR anopheline) and (Thailand OR Thai OR Siam)” were used to identify relevant studies in each database (Additional file 1: Table S1). Studies that focused on the identification of *Plasmodium* spp. in *Anopheles* mosquitoes were selected. The reference lists of the included studies were also screened to ensure that relevant studies were not missed. The search included only studies that were published in English between 1945 and 2021.

### Eligibility criteria and study selection

Studies were selected using the PICO method. The elements of this method are as follows: P: Participants. The participants included in the study were *Anopheles* mosquitoes in Thailand. I: Intervention. No intervention was applied in the present study. C: Comparator. No comparator was used in the present study. O: Outcome. The outcome of interest was the presence of *Plasmodium* spp. in any stage that was identified in *Anopheles* mosquitoes. Thus, the inclusion criteria were composed of cross-sectional studies that identified *Plasmodium* spp. among *Anopheles* mosquitoes collected in Thailand. The exclusion criteria were studies with incomplete data for extraction, studies for which the full text was unavailable, reviews or systematic reviews, in vitro studies, papers describing the development of assays, and letters to the editor/comments/editorials. Two authors (CS and MK) independently screened the titles and abstracts and selected studies based on the eligibility criteria. First, the titles and abstracts generated by the electronic search were checked. Second, the full texts were examined, and studies that did not meet the eligibility criteria were excluded, with the reasons recorded. Any differences in study selection between the two authors were resolved by mutual consensus.

### Data extraction and quality assessment

Pilot data extraction tables were used to collect information from each included study. The following information was collected: name of the first author, year of publication, study sites, season and time at which mosquitoes were collected, mosquito collection methods, mosquito species, number of mosquitoes, methods of *Plasmodium* detection, *Plasmodium* identified, and stage of *Plasmodium* spp. The quality of the studies included was determined using the Strengthening the Reporting of Observational Studies in Epidemiology (STROBE) statement, which includes 22 parameters [32]. The quality of each study was assessed as high quality (> 75%), moderate quality (50–75%), or low quality (< 50%). High-quality and moderate-quality studies were included in the systematic review. Low-quality studies were excluded.

### Data syntheses

The proportion of *Anopheles* mosquitoes collected in the studies, *Anopheles* vectors positive for *Plasmodium* species, and *Plasmodium* spp. identified in *Anopheles* mosquitoes are presented as frequencies and percentages. The pooled prevalence of *Plasmodium* species among *Anopheles* mosquitoes and primary vectors (*An. dirus*, *An. minimus*, and *An. maculatus*) was estimated using a random-effects meta-analysis using the DerSimonian and Laird method [33]. The proportions from each study were pooled using logit transformation, and back transformation to a proportion was performed using generalized linear mixed models (GLMMs). The individual study weights were not calculated by the GLMMs. The meta-analyses of proportion studies were conducted using the command “metaprop_one” in Stata version 14.0 software (StataCorp LLC, College Station, TX, USA) as described previously [34]. Forest plots were used to depict the study-specific proportions with 95% exact confidence intervals and overall pooled estimates with 95% Wald confidence intervals. The Chi-square statistic of the likelihood ratio test was used to identify the presence of significant heterogeneity when the *P*-value was less than 0.05. As the individual study weights were not calculated by the GLMMs, publication bias assessment was not performed in the present study. For the meta-analyses of proportion studies with low proportion outcomes, funnel plots were not used, as they are ineffective at detecting potential publication bias [35].

## Results

### Search results

**Fig. 1:**
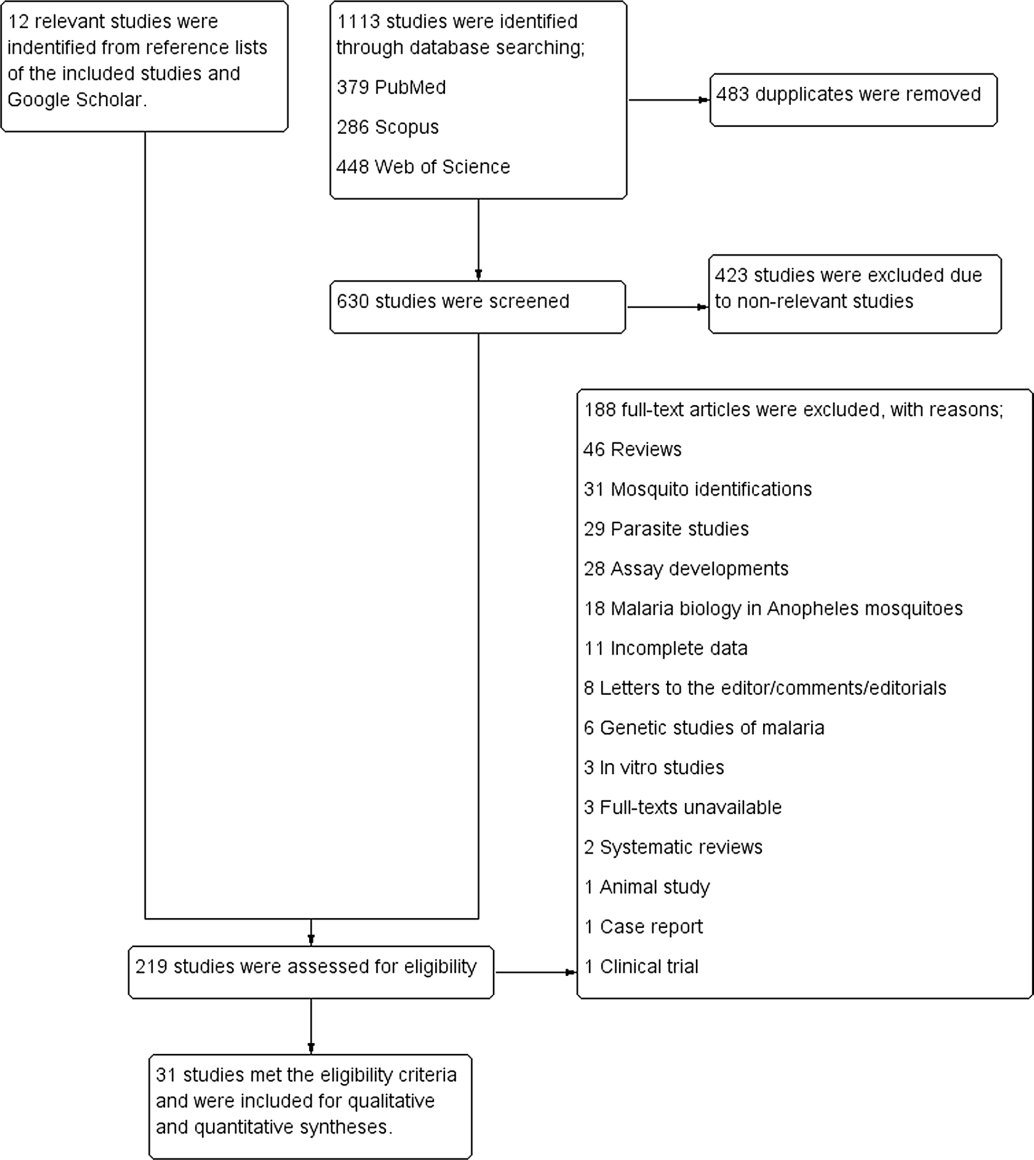
Study flow diagram

A total of 1113 candidate studies were identified through PubMed (379 studies), Web of Science (448 studies), and Scopus (286 studies). After 483 duplicates were removed, 630 studies were screened for titles and abstracts, and 207 studies remained for further full-text examination. The examination of the full text of the studies identified 19 studies [15–17, 19, 20, 23, 36–48] that met the eligibility criteria. One hundred and eighty-eight studies were excluded for specific reasons: 46 reviews, 31 mosquito identifications, 29 parasite studies, 28 assay developments, and 18 malaria biology in *Anopheles* mosquitoes; 11 for incomplete data; eight letters to the editor/comments/editorials; six genetic studies of malaria; three in vitro studies; three studies for which the full text was unavailable; two systematic reviews; one animal study; one case report; and one clinical trial. An additional 12 relevant studies [49–60] from the reference lists of the included studies and Google Scholar were examined for full texts. All of them met the eligibility criteria and were included in the systematic review. Overall, 31 studies [15–17, 19, 20, 23, 36–60] were included in the present systematic review (Fig. 1).

### Characteristics of the studies

**Fig. 2:**
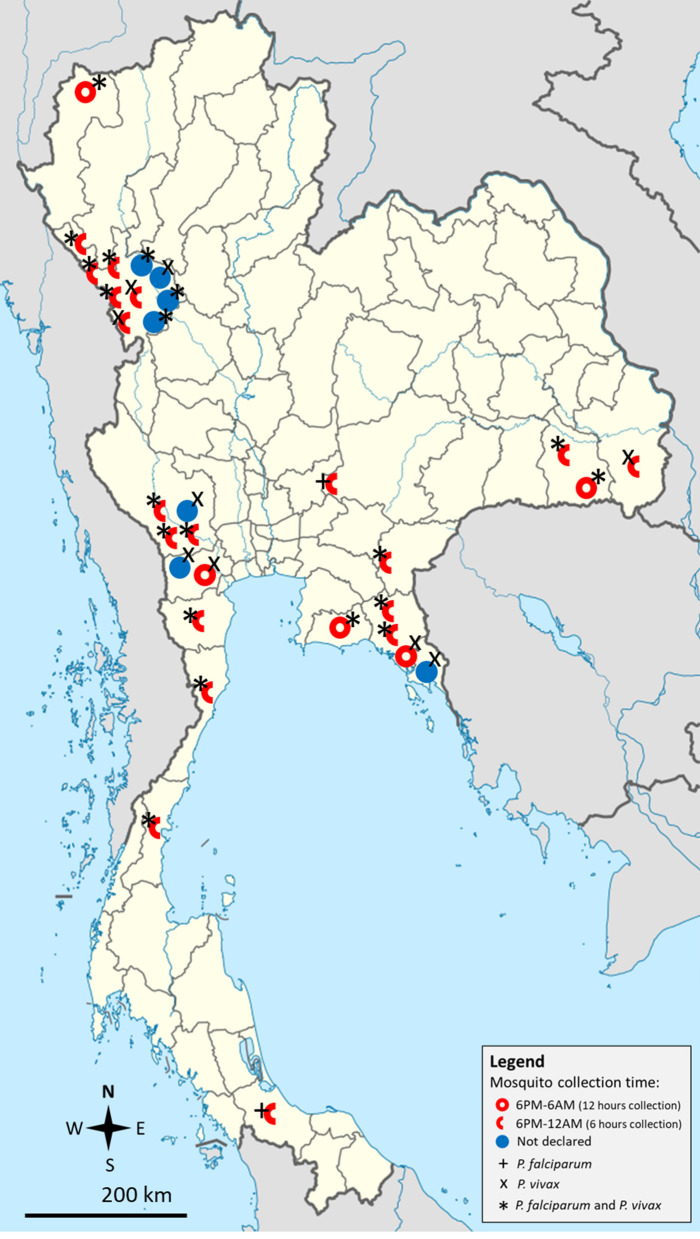
Geographic distribution of mosquito identification studies with isolated malaria-causing *Plasmodium* spp. in Thailand. Map of Thailand (Thailand location map.svg) was sourced license-free from Wikimedia commons: https://commons.wikimedia.org/w/index.php?search=thailand+map&title=Special:MediaSearch&go=Go&type=image

The characteristics of the included studies are shown in Table 1. The studies were published in 1970–2000 (13 studies, 41.9%), 2001–2010 (seve studies, 22.6%), and 2011–2021 (11 studies, 35.5%). The studies were conducted in western Thailand (14 studies, 45.2%), eastern Thailand (4 studies, 12.9%), northeastern Thailand (four studies, 12.9%), and other parts of Thailand (nine studies, 29.0%). Most of the studies were conducted in Tak (nine studies, 29%) and Kanchanaburi (three studies, 9.68%). For mosquito collection, the studies used human landing collection (17 studies, 54.8%), human landing collection/animal-baited collection (six studies, 19.4%), light trap (two studies, 6.5%), human landing collection/animal-baited collection/light trap (two studies, 6.5%), human landing collection/light trap (one study, 3.22%), animal-baited collection/light trap (one study, 3.22%), and prokopack aspiration (one study, 3.22%), and one study did not specify the mosquito collection method (one study, 3.22%). Most of the studies collected mosquitoes between 18:00 and 06:00 (48.4%) and between 18:00 and 24:00 (12.9%). The details of the studies are shown in Additional file 2: Table S2. The distributions of *Plasmodium*-positive mosquitoes are shown in Fig. 2.

**Table 1:** Characteristics of the included studies

Parameters	Number of studies (%)
Publication years	
1970–2000	13 (41.9%)
2001–2010	7 (22.6%)
2011–2021	11 (35.5%)
Study locations	
Western Thailand	14 (45.2%)
Tak	9
Kanchanaburi	3
Ratchaburi	2
Northern Thailand	2 (6.5%)
Chiang Mai	1
Mae Hong Son	1
Eastern Thailand	4 (12.9%)
Chantaburi	2
Sa Kaeo	1
Rayong	1
Northeastern Thailand	4 (12.9%)
Nakhon Ratchasima	2
Ubon Ratchathani	2
Eastern and northeastern Thailand	2 (6.5%)
Sa Kaeo/Chanthaburi/Sisaket	1
Chanthaburi/Sisaket	1
Northeastern and southern Thailand	1 (3.22%)
Nakhon Ratchasima/Songkhla	1
Western and southern Thailand	1 (3.22%)
Phetchaburi/Prachuap Khiri Khan/Chumpon	1
Northern and western Thailand	1 (3.22%)
Tak/Mae Hong Son	1
Eastern and southern Thailand	1 (3.22%)
Chanthaburi/Surat Thani	1
Eastern and western Thailand	1 (3.22%)
Kanchanaburi/Trat	1
Mosquito collection method	
Human landing collection	17 (54.8%)
Human landing collection/animal-baited collection	6 (19.4%)
Light trap	2 (6.5%)
Human landing collection/animal-baited collection/light trap	2 (6.5%)
Human landing collection/light trap	1 (3.2%)
Animal-baited collection/light trap	1 (3.2%)
Prokopack aspiration	1 (3.2%)
Not specified	1 (3.2%)
Time for mosquito collection	
18:00–06:00	15 (48.4%)
18.00–24.00	4 (12.9%)
18:00–04:50	1 (3.2%)
18:30–24:00	1 (3.2%)
18:30–05:00	1 (3.2%)
18:00–05:00	1 (3.2%)
19:00–04:50	1 (3.2%)
6:00–9:30 and 4:30–8:30	1 (3.22%)
Not specified	6 (19.4%)

### Quality of the studies

The quality of the included studies was assessed using the STROBE checklist (Additional file 3: Table S3). For the included studies identified from the three databases, most of the studies (14/19, 73.7%) [20, 23, 37–42, 44–48, 51] were of moderate quality, whereas four (21.1%) were of high quality [15, 16, 36, 43] and one study (5.26%) [19] was of low quality. For the included studies identified from the reference lists and Google Scholar, most of the studies (9/12, 75%) [49, 51, 52, 54, 56–60] were of moderate quality, whereas three of them (25%) [50, 53, 55] were of high quality. Overall, seven studies (22.58%) were of high quality. Twenty-three studies (74.19%) were of moderate quality. Only one low-quality study [19], a short report, was included in the systematic review because it contained essential data on *Plasmodium* infection in primary *Anopheles* vectors.

### *Anopheles* mosquitoes collected in the studies

The list of the *Anopheles* mosquitoes collected from the studies is shown in Table 2. The total number of *Anopheles* mosquitoes collected from all studies was 126,025. Of those *Anopheles* mosquitoes, 100,910 were identified to species. Most of the *Anopheles* species identified were *An. minimus* (40.16%), *An. maculatus* (16.59%), *An. epiroticus* (9.18%), *An. sawadwongporni* (6.35%), *An. barbirostris* (5.30%), *An. dirus* (4.91%), *An. peditaeniatus* (Leicester) (3.66%), *An. aconitus* (2.69%), *An. philippinensis* Ludlow (1.34%), *An. nivipes* (Theobald) (1.27%), *An. campestris* (1.26%), and *An. karwari* (James) (1.03%). The studies by Nosten et al. [55] and Brusich et al. [36] did not specify the *Anopheles* species collected in their study, whereas a study by Carrara et al. [53] collected only *An. minimus*, *An. maculatus*, and *An. dirus*. Another study by Sriwichai et al. [44] collected only *An. minimus* and *An. maculatus*.

**Table 2:** *Anopheles* mosquitoes collected from the included studies

Mosquito species	No. of *Anopheles* mosquitoes	%
*An. minimus* Theobald	40,984	40.61
*An. maculatus* Theobald	16,742	16.59
*An. epiroticus* Linton & Harbach	9260	9.18
*An. sawadwongporni* Rattanarithikul & Green	6593	6.53
*An. barbirostris* van der Wulp	5352	5.30
*An. dirus* Peyton & Harrison	4956	4.91
*An. peditaeniatus* (Leicester)	3690	3.66
*An. aconitus* Dönitz	2712	2.69
*An. philippinensis* Ludlow	1354	1.34
*An. nivipes* (Theobald)	1285	1.27
*An. campestris* Reid	1275	1.26
*An. karwari* (James)	1043	1.03
*An. tessellatus* Theobald	1007	1.00
*An. annularis* van der Wulp	852	0.84
*An. umbrosus* (Theobald)	819	0.81
*An. kochi* Dönitz	519	0.51
*An. pseudowillmori* (Theobald)	464	0.46
*An. hyrcanus* (Pallas)	431	0.43
*An. culicifacies* Giles	297	0.29
*An. varuna* Iyengar	270	0.27
*An. vagus* Dönitz	215	0.21
*An. jamesii* Theobald	210	0.21
*An. rampae* Harbach & Somboon	142	0.14
*An. baimaii* Sallum & Peyton	86	0.09
*An. balabacensis* Baisas	84	0.08
*An. pseudojamesi* Strickland & Chowdhury	79	0.08
*An. splendidus* Koidzumi	52	0.05
*An. nitidus* Harrison, Scanlon & Reid	45	0.04
*An. nigerrimus* Giles	24	0.02
*An. subpictus* Grassi	18	0.02
*An. dravidicus* Christophers	12	0.01
*An. greeni* Rattanarithikul & Harbach	8	0.01
*An. indefinitus* (Ludlow)	7	0.01
*An. hodgkini* Reid	6	0.01
*An. notanandai* Rattanarithikul & Green	5	0.00
*An. argyropus* (Swellengrebel)	4	0.00
*An. barbumbrosus* Strickland & Chowdhury	3	0.00
*An. letifer* Sandosham	2	0.00
*An. jeyporiensis* James	1	0.00
*An. sinensis* Wiedemann	1	0.00
*An. willmori* (James)	1	0.00
Total (species were specified)	100,910	100
*An. minimus, An. maculatus, and An. dirus* (total number was reported)	22,821	
*An. minimus* and *An. maculatus* (total number was reported)	798	
*Anopheles* spp. (species were not specified)	1496	
Total mosquitoes in all studies	126,025	

### *Anopheles* vectors for *Plasmodium* species

**Fig. 3:**
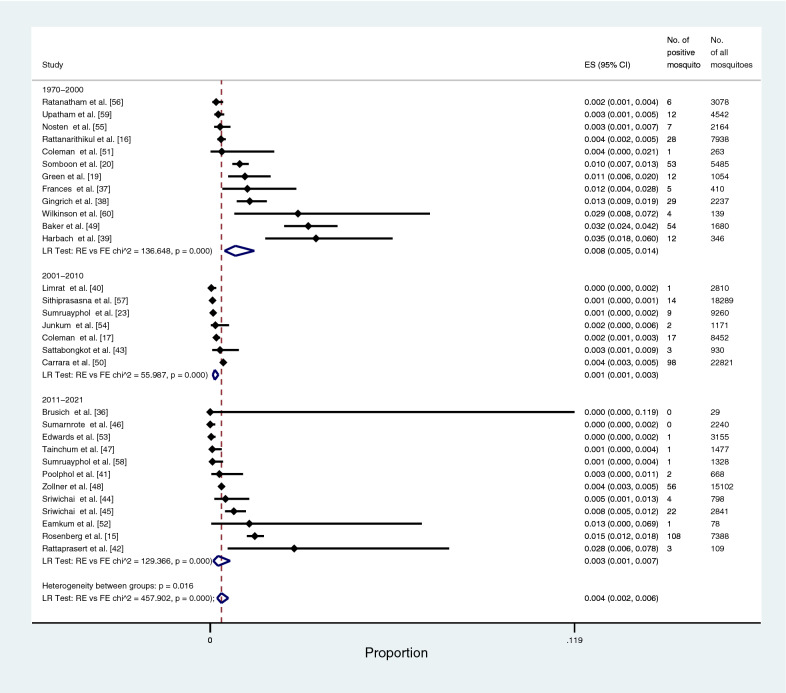
The pooled prevalence of *Plasmodium* spp. in all vectors. ES, prevalence estimate; 95% CI: confidence interval

The prevalence of the *Plasmodium* species identified in all *Anopheles* vectors was estimated using the data from 31 studies [15–17, 19, 20, 23, 36–60]. This meta-analysis showed that the pooled prevalence of *Plasmodium* species identified in all *Anopheles* vectors among studies conducted during 1970–2000 was 1.7% (95% CI: 0.5–6.3%, Chi-square: 347.91, *P* < 0.001, 12 studies). The pooled prevalence of *Plasmodium* species identified in all *Anopheles* vectors among studies conducted during 2001–2010 was 0.11% (95% CI: 0.1–0.3%, Chi-square: 56, *P* < 0.001, 7 studies), and that among studies conducted during 2011–2021 was 0.2% (95% CI: 0.1–0.7%, Chi-square: 131, *P* < 0.001, 12 studies). Overall, the pooled prevalence of *Plasmodium* species identified in all *Anopheles* vectors was 0.5% (95% CI: 0.2–1.1%, Chi-square: 738.6, *P* < 0.001, 31 studies; Fig. 3).

**Fig. 4:**
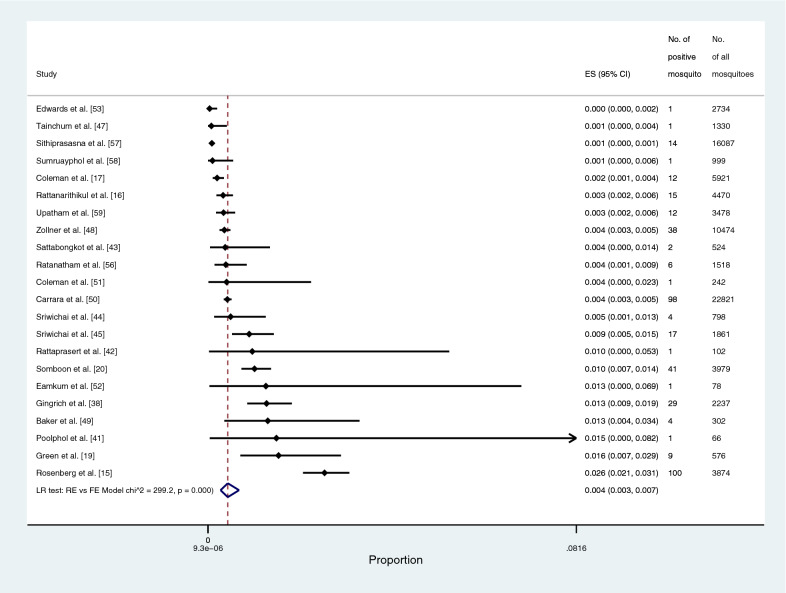
The pooled prevalence of *Plasmodium* spp. in primary vectors (*An. minimus*, *An. maculatus*, *An. dirus*). ES, prevalence estimate; 95% CI: confidence interval

**Fig. 5:**
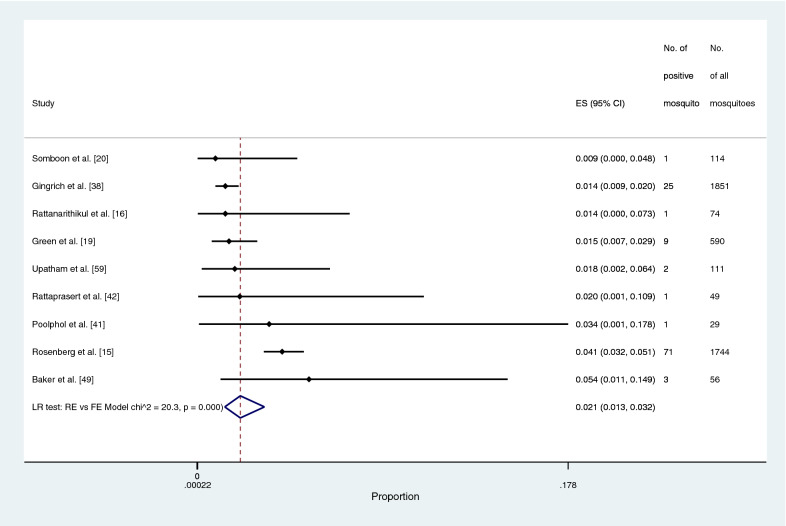
The pooled prevalence of *Plasmodium* spp. in *An. dirus*. ES, prevalence estimate; 95% CI: confidence interval

**Fig. 6:**
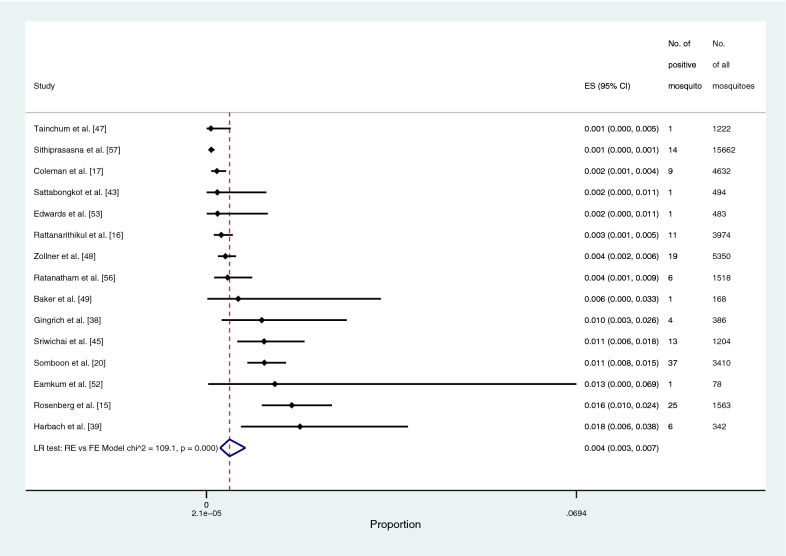
The pooled prevalence of *Plasmodium* spp. in *An. minimus*. ES, prevalence estimate; 95% CI: confidence interval

**Fig. 7:**
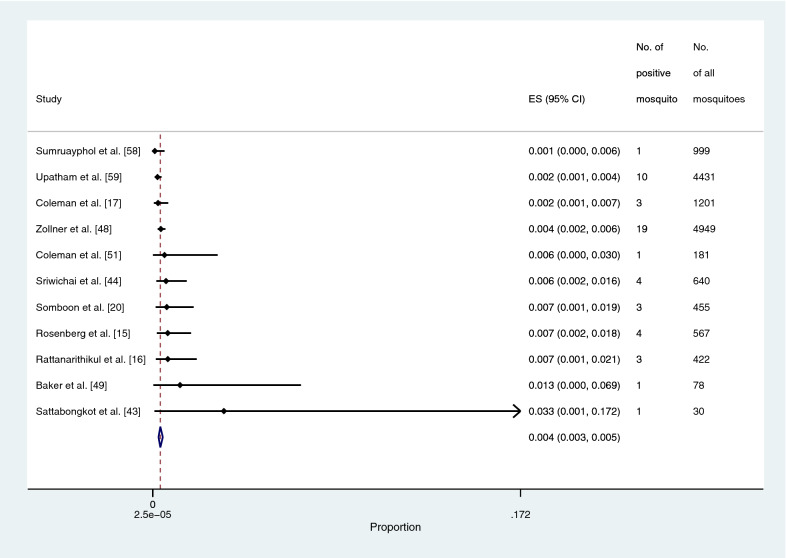
The pooled prevalence of *Plasmodium* spp. in *An. maculatus*. ES, prevalence estimate; 95% CI: confidence interval

The prevalence of *Plasmodium* species identified in the main *Anopheles* vectors (*An. dirus*, *An. maculatus*, and *An. minimus*) was estimated using the data from 22 studies [15–17, 19, 20, 38, 41–45, 47–53, 56–59]. The pooled prevalence of *Plasmodium* species identified in the main *Anopheles* vectors was 0.4% (95% CI: 0.3–7%, Chi-square: 299.2, *P* < 0.001, 22 studies; Fig. 4). The pooled prevalence of *Plasmodium* species identified in *An. dirus* was 2.1% (95% CI: 1.3–3.2%, Chi-square: 20.3, *P* < 0.001, nine studies; Fig. 5). The pooled prevalence of *Plasmodium* species identified in *An. minimus* was 0.4% (95% CI: 0.3–0.7%, Chi-square: 109.1, *P* < 0.001, 15 studies; Fig. 6). The pooled prevalence of *Plasmodium* species identified in *An. maculatus* was 0.4% (95% CI: 0.3–0.5%, Chi-square: 0.1, *P*: 0.385, 11 studies; Fig. 7).

Among the particular *Anopheles* species identified (*n* = 123,286), 566 *Anopheles* mosquitoes (0.46%) were positive for *Plasmodium* species The *Anopheles* species that tested positive for *Plasmodium* were *Anopheles hodgkini* Reid, *Anopheles nigerrimus* Giles, *Anopheles balabacensis* Baisas, *An. dirus*, *Anopheles annularis* van der Wulp, *Anopheles kochi* Dönitz, *Anopheles vagus* Dönitz, *An. baimaii*, *Anopheles hyrcanus* (Pallas), *An. karwari*, *An. pseudowillmori*, *An. aconitus*, *An. sawadwongporni*, *An. barbirostris*, *An. minimus*, *Anopheles varuna* Iyengar, *An. maculatus*, *An. nivipes*, *An. peditaeniatus*, *An. campestris*, *An. philippinensis*, and *An. epiroticus* (Table 3). The highest proportions of *Plasmodium*-positive mosquitoes were identified as *An. hodgkini* (2/6, 33.3%), *An. nigerrimus* (2/24, 8.33%), *An. balabacensis* (4/84, 4.76%), *An. dirus* (114/4956, 2.3%), *An. annularis* (16/852, 1.88%), *An. kochi* (8/519, 1.54%), *An. vagus* (3/215, 1.40%), and *An. baimaii* (1/86, 1.16%). Other *Anopheles* vectors for *Plasmodium* species present in a smaller proportion included *An. karwari* (0.67%), *An. pseudowillmori* (0.65%), *An. aconitus* (0.55%), *An. sawadwongporni* (0.47%), *An. barbirostris* (0.45%), *An. minimus* (0.38%), *An. varuna* (0.37%), *An. maculatus* (0.3%), *An. hyrcanus* (0.23%), *An. nivipes* (0.16%), *An. peditaeniatus* (0.16%), *An. campestris* (0.16%), *An. philippinensis* (0.15%), and *An. epiroticus* (0.1%). Some of the *Anopheles* species (*n* = 2739) were negative for *Plasmodium* species.

**Table 3:** *Anopheles* vectors positive for *Plasmodium* species in Thailand

Mosquito species	No. of *Anopheles*	No. of positive	% positive
*An. hodgkini*	6	2	33.33
*An. nigerrimus*	24	2	8.33
*An. balabacensis*	84	4	4.76
*An. dirus*	4956	114	2.30
*An. annularis*	852	16	1.88
*An. kochi*	519	8	1.54
*An. vagus*	215	3	1.40
*An. baimaii*	86	1	1.16
*An. karwari*	1043	7	0.67
*An. pseudowillmori*	464	3	0.65
*An. aconitus*	2712	15	0.55
*An. sawadwongporni*	6593	31	0.47
*An. barbirostris*	5352	24	0.45
*An. minimus*	40,984	154	0.38
*An. varuna*	270	1	0.37
*An. maculatus*	16,742	50	0.30
*An. hyrcanus*	431	1	0.23
*An. nivipes*	1285	2	0.16
*An. peditaeniatus*	3690	6	0.16
*An. campestris*	1275	2	0.16
*An. philippinensis*	1354	2	0.15
*An. epiroticus*	9260	9	0.10
*Anopheles* spp. (species were not identified)	1470	7	0.48
Total (species were identified)	99,667	464	0.47
*An. minimus*, *An. maculatus*, and *An. dirus* (total number was reported)	22,821	98	0.43
*An. minimus* and *An. maculatus* (total number was reported)	798	4	0.50
All mosquitoes	123,286	566	0.46
*Anopheles* spp. negative for *Plasmodium* species	2739		
Total mosquitoes in all studies	126,025		

### Proportion of *Plasmodium* species identified in each *Anopheles* vector

Table 4 shows the proportion of *Plasmodium* species identified in *Anopheles* vectors. High proportions of *P. falciparum* were identified in *An. annularis* (8.50%), *An. kochi* (6.03%), and *An. barbirostris* (5.03%). High proportions of *P. vivax* were identified in *An. hodgkini* (33.3%), *An. aconitus* (28.6%), and *An. nivipes* (25.0%). High proportions of *P. falciparum/P. vivax* were identified in *An. nigerrimus* (14.3%), *An. vagus* (2.88%), and *An. peditaeniatus* (2.47%). High proportions of *P. falciparum*/*P. vivax*/mixed infection were identified in *An. dirus* (7.27%). High proportions of *Plasmodium* spp. (species not identified) were *An. balabacansis* (4.76%) and *An. dirus* (4.07%).

**Table 4:** Proportion of *Plasmodium* species identified in each *Anopheles* mosquito in Thailand

*Plasmodium* species	*Anopheles* mosquitoes	No. of *Anopheles*	No. of positive	Percentage
*P. falciparum*	*An. annularis*	153	13	8.50
	*An. kochi*	116	7	6.03
	*An. barbirostris*	199	10	5.03
	*An. baimaii*	53	1	1.89
	*An. dirus*	371	5	1.35
	*An. aconitus*	948	11	1.16
	*An. minimus*	3410	37	1.09
	*An. maculatus*	955	7	0.73
	*An. sawadwongporni*	706	5	0.71
	*An. nivipes*	211	1	0.47
*P. falciparum*/*P. vivax*	*An. nigerrimus*	14	2	14.29
	*An. vagus*	104	3	2.88
	*An. peditaeniatus*	162	4	2.47
	*An. dirus*	1907	28	1.47
	*An. pseudowillmori*	384	3	0.78
	*An. sawadwongporni*	5001	25	0.50
	*An. maculatus*	4949	19	0.38
	*An. barbirostris*	1255	5	0.40
	*An. minimus*	30,346	68	0.22
	*An. epiroticus*	9260	9	0.10
*P. falciparum/P. vivax*/Mixed infection	*An. dirus*	110	8	7.27
	*An. annularis*	431	3	0.70
	*An. maculatus*	640	4	0.63
*P. vivax*	*An. hodgkini*	6	2	33.33
	*An. aconitus*	7	2	28.57
	*An. nivipes*	4	1	25.00
	*An. karwari*	54	2	3.70
	*An. dirus*	65	2	3.08
	*An. philippinensis*	97	1	1.03
	*An. hyrcanus*	105	1	0.95
	*An. varuna*	113	1	0.88
	*An. kochi*	159	1	0.63
	*An. sawadwongporni*	182	1	0.55
	*An. minimus*	3459	18	0.52
	*An. barbirostris*	1694	8	0.47
	*An. campestris*	478	2	0.42
	*An. maculatus*	2411	6	0.25
*Plasmodium* spp.	*An. balabacansis*	84	4	4.76
	*An. dirus*	1744	71	4.07
	*An. minimus*	3081	31	1.01
	*An. philippinensis*	132	1	0.76
	*An. karwari*	821	5	0.61
	*An. maculatus*	4998	14	0.28
	*An. aconitus*	1025	2	0.20
	*An. peditaeniatus*	2179	2	0.09
	*An. barbirostris*	1317	1	0.08

## Discussion

The present systematic review showed that *An. minimus* (40.16%), *An. maculatus* (16.59%), and *An. epiroticus* (9.18%) were the most common *Anopheles* mosquitoes identified in the studies included. The differences in the proportions of mosquito species might be due to the variations in the local environment, such as differences in the study site, seasonal, biology, and behavior of each species. Although *An. minimus*, *An. maculatus*, and *An. epiroticus* were the main mosquitoes identified in the studies, these mosquitoes harbored *Plasmodium* spp. at only 0.38%, 0.3%, and 0.1%, respectively. However, it is well recognized that several of the important malaria vectors, as well as other *Anopheles* species in Thailand, are members of closely related sibling species. Thus, entomologists classify them into complexes or groups [9]. Although illustrated morphological keys have been published for the identification of both female adult and larval stages of *Anopheles* mosquitoes in Thailand [11], the identification of *Anopheles* specimens based exclusively on morphological characteristics is questionable and potentially leads to misidentification [9].

The three main malaria vectors in Thailand are *An. minimus*, *An. maculatus*, and *An. dirus*, as previously reported by the Division of Vector Borne Disease, Ministry of Public Health, Thailand [61]. The present pooled analyses showed that 0.4%, 0.4%, and 2.1%, respectively, of these *Anopheles* mosquitoes harbored *Plasmodium* parasites. Therefore, meta-analysis in the present study suggested that there was a very low prevalence of *Plasmodium* spp. in the main *Anopheles* mosquitoes in Thailand. The success of the implementation of vector control tools, such as LLINs and IRS, across Thailand, has significantly reduced malaria transmission from 149,586 cases in 2000 [62] to 4473 cases in 2020 [5], thus possibly contributing to the low prevalence of *Plasmodium* in field populations. Other factors such as a change in behavior of *Anopheles* mosquitoes due to elimination efforts [63], climate change, and urbanization [64] might also have affected the EIR in terms of both human biting rate and SRs. To determine the annual EIR and to consequently evaluate vector control interventions, we recommend that a relatively large number of mosquito specimens (more than 1000 mosquitoes) are collected for an accurate analysis of SRs in each locality, preferably as a part of a long-term study. To achieve malaria eradication in Thailand by 2024 [5], further *Anopheles* studies should be performed in provinces along the international borders, such as the Tak, Kanchanaburi, Chanthaburi, Ubon Ratchathani, and Yala provinces, in which the disease is endemic (Fig. 2) [5, 65].

To identify where malaria transmission occurs, reliable estimations of the proportions of infective *Anopheles* mosquitoes, as reflected by the presence of sporozoites in the salivary gland, are needed [66, 67]. Several techniques have been used to quantify sporozoites in mosquitoes. Dissection and microscopic examination of the salivary glands is considered the “gold standard” method, but this approach is labor intensive and impractical in the field [68]. Enzyme-linked immunosorbent assay to detect circumsporozoite proteins (CSP-ELISA) is another widely used technique [69]. However, CSP-ELISA has been shown to give false positive results, thus overestimating the real SR [70]. Molecular-based methods have been developed to improve sensitivity and specificity [71]. In our analysis, 19 studies (61.30%) used CSP-ELISA as a means of *Plasmodium* detection. Six studies (19.35%) used dissection of the salivary gland and gut. It is possible that the limitations of the dissection and CSP-ELISA methods used in the studies could have affected the estimation of sporozoite infection. Six studies (19.35%) used polymerase chain reaction (PCR)-based techniques. Sumruayphol et al. [23] performed nested PCR and real-time PCR on 9260 *An. epiroticus* specimens and found only six mosquitoes infected with *P. falciparum* and three with *P. vivax*. In another study, Tainchum et al. [47] also used real-time PCR for *Plasmodium* detection in 1090 *An. minimus* specimens and found only one positive sample. These studies suggested that even using methods with high sensitivity, the prevalence of *Plasmodium* species in *Anopheles* mosquitoes was very low, suggesting that the prevalence of parasite vectors in Thailand was genuinely low. Other techniques such as rapid dipstick immunochromatographic assays (Vec-Test™ Malaria) [72] and near-infrared spectroscopy [73] have also been developed for *Plasmodium* detection in *Anopheles* mosquitoes. Overall, to avoid the overestimation of SR and the EIR, it is highly recommended that all positive CSP-ELISA samples be reanalyzed or the results confirmed by performing *Plasmodium*-specific PCR [70].

*Anopheles dirus* had the highest pooled prevalence of *Plasmodium* species identified (2.1%) in our analysis. However, it should also be noted that the high prevalence of infection does not necessarily translate to the species being the main vector. Other factors also play a crucial role in the importance of primary vectors, for instance, the species must often be abundantly present and prefer to feed on humans [10]. Previous studies also reported relatively low numbers of collected *An. dirus* specimens (ranging from 10–78 mosquitoes/location) recently [41, 42, 45–47, 53]. Therefore, it is vital to assess transmission indicators (e.g., EIR) to determine the importance of each vector species. The prevalence of *Plasmodium* species identified in *An. dirus* decreased from 5% in 1987 [49] to 1% in 1990 [38]. However, the prevalence of *Plasmodium* species identified in *An. dirus* increased to 4% in 1990 [15] and decreased to 1–3% during 1991–2017 [16, 19, 20, 41, 42]. The yearly trend results were heterogeneous, and differences in study sites had to be considered. The pooled prevalence of *Plasmodium* species identified in *An. minimus* and *An. maculatus* was the same at 0.4%. These results suggest that the likelihood of finding an infected wild *An. minimus* or *An. maculatus* is lower than that of finding an infected *An. dirus*. The reasons for this observation remain to be investigated. However, several factors influencing vectorial capacity and competence have been documented, including mosquito longevity, the duration of sporogonic development, and the susceptibility or resistance of the vector to *Plasmodium* [74]. A previous study of three laboratory strains of *An. dirus*, *An. minimus*, and *An. sawadwongporni* showed similar susceptibility to *P. vivax* infection using an artificial feeding system [75]. These laboratory-raised mosquitoes are highly inbred and may be genetically dissimilar to the originally sampled population [76]. In our analysis, natural *Plasmodium* infection in wild *Anopheles* mosquitoes was also considerably different among populations and species. In another study, large differences in *P. falciparum* infection were observed in a wild population of *Anopheles gambiae* Giles in West Africa [77]. Therefore, it is not surprising to find differences in *Plasmodium* prevalence in the diverse field populations in Thailand.

The present study had some limitations. First, the studies that were included for systematic review were not performed in all areas in which malaria cases have been reported. There are missing mosquito data from the Thailand-Malaysia border, where high levels of malaria have been reported. Hence, the systematic review did not represent the overall prevalence in Thailand. Second, the majority of the studies (14 studies, 45.16%) used in our analysis used only morphological keys for species identification, and an additional 12 studies (38.71%) did not indicate which identification method was used. Therefore, some of the *Anopheles* specimens in these studies were only classified into complexes or groups. We therefore simply reported and analyzed the species data using the information presented in the studies. Only five studies (16.13%) used molecular techniques to confirm the species of the *Anopheles* specimens after the initial morphological identification into complexes or groups. To reflect the true prevalence of *Plasmodium* in each *Anopheles* species, particularly the primary and secondary malaria vectors, species confirmation using molecular techniques should also be performed. Third, it has been demonstrated that a positive SR may coincide with peak mosquito populations [16, 20, 48]. Thus, the prevalence of *Plasmodium* infection in each mosquito species could also be varied depending on season and mosquito abundance. In our analysis, only cross-sectional studies were included and we did not attempt to factor seasonal variation, as some included studies did not report seasonal information of positive *Plasmodium* infection specimens. However, is it possible that in any of the included studies, data for one species might have been collected during high transmission, whereas data for other species might have been collected during low transmission seasons, which might be a source of overestimation and underestimation of the importance of different vectors. Fourth, we included studies that identified *Anopheles*-harbored sporozoites and also oocysts of *Plasmodium* spp. As these stages are different indicators and may have different interpretations, we used the oocyst formation rate to study infection in the vector, that is, to show the susceptibility of the vector to infection; however, it does not indicate the importance of the vector in transmission. Therefore, the prevalence of *Plasmodium* spp. in *Anopheles* mosquitoes indicates the infection rates rather than the transmission capability. Finally, there are few synthesized studies, which are then subdivided into smaller subgroups for the purpose of comparing differences in mosquito species.

## Conclusions

This systematic review confirmed the relatively low prevalence of *Plasmodium* species in wild *Anopheles* mosquitoes in Thailand. *Anopheles dirus* was likely to be the predominant species harboring *Plasmodium* species. However, the measurement of the number of sporozoites must be performed with caution to avoid overestimating the extent of *Plasmodium* infection. An accurate estimation of the EIR using a standardized parasite detection technique would also require the use of a relatively large number of *Anopheles* specimens to assess the impact of vector control interventions. The results of the present study also serve to identify potential vectors for malaria as a basis for further detailed studies. With this information, more intensive mosquito studies should be undertaken in several areas of Thailand to explore the prevalence of *Plasmodium* species in *Anopheles* mosquitoes as a basis for the development of vector control strategies.

## Supplementary Information

**Additional file 1: Table S1.** Search terms

**Additional file 2: Table S2.** Details of the included studies

**Additional file 3: Table S3.** Quality of the included studies

## Data Availability

All data relating to the present study are available in this manuscript.

## Abbreviations

EIR: Entomological inoculation rate
GLMM: Generalized linear mixed models
SR: Sporozoite rate

## References

[1] WHO (2021). World malaria report 2021.

[2] Cui L, Cao Y, Kaewkungwal J, Khamsiriwatchara A, Lawpoolsri S, Soe TN, Manguin S, Dev V (2018). Malaria elimination in the Greater Mekong Subregion: challenges and prospects. Towards malaria elimination - A leap forward.

[3] Corbel V, Nosten F, Thanispong K, Luxemburger C, Kongmee M, Chareonviriyaphap T (2013). Challenges and prospects for dengue and malaria control in Thailand. Southeast Asia Trends Parasitol.

[4] Suwonkerd W, Ritthison W, Ngo CT, Tainchum K, Bangs MJ, Chareonviriyaphap T, Manguin S (2013). Vector biology and malaria transmission in Southeast Asia, *Anopheles* mosquitoes. New insights into malaria vectors.

[5] Bureau of Vector-Borne Diseases (2020). Annual Report 2020.

[6] WHO (2018). World Malaria Report 2018.

[7] Bureau of Vector-Borne Diseases (2019). Guide to malaria elimination for Thailand’s local administrative organizations and the health network.

[8] Durnez L, Coosemans M (2013). Residual transmission of malaria: an old issue for new approaches.

[9] Tainchum K, Kongmee M, Manguin S, Bangs MJ, Chareonviriyaphap T (2015). *Anopheles* species diversity and distribution of the malaria vectors of Thailand. Trends Parasitol.

[10] Tananchai C, Manguin S, Bangs MJ, Chareonviriyaphap T (2019). Malaria vectors and species complexes in Thailand: Implications for vector control. Trends Parasitol.

[11] Rattanarithikul R, Harrison BA, Harbach RE, Panthusiri P, Coleman RE (2006). Illustrated keys to the mosquitoes of Thailand. IV. Anopheles. Southeast Asian J Trop Med Public Health.

[12] Sinka ME, Bangs MJ, Manguin S, Chareonviriyaphap T, Patil AP, Temperley WH (2011). The dominant *Anopheles* vectors of human malaria in the Asia-Pacific region: occurrence data, distribution maps and bionomic précis. Parasit Vectors.

[13] Harbach RE, Manguin S (2013). The phylogeny and classification of *Anopheles*, *Anopheles* mosquitoes. Anopheles mosquitoes - New insights into malaria vectors.

[14] Harbach RE, Rattanarithikul R, Harrison BA (2017). *Anopheles prachongae*, a new species of the Gigas Complex of subgenus *Anopheles* (Diptera: Culicidae) in Thailand, contrasted with known forms of the complex. Zootaxa.

[15] Rosenberg R, Andre RG, Somchit L (1990). Highly efficient dry season transmission of malaria in Thailand. Trans R Soc Trop Med Hyg.

[16] Rattanarithikul R, Konishi E, Linthicum KJ (1996). Detection of *Plasmodium vivax* and *Plasmodium falciparum* circumsporozoite antigen in anopheline mosquitoes collected in southern Thailand. Am J Trop Med Hyg.

[17] Coleman RE, Sithiprasasna R, Kankaew P, Kiaattiut C, Ratanawong S, Khuntirat B (2002). Naturally occurring mixed infection of *Plasmodium vivax* VK210 and *P*. *vivax* VK247 in *Anopheles* mosquitoes (Diptera: Culicidae) in western Thailand. J Med Entomol.

[18] Gould DJ, Esah S, Pranith U (1967). Relation of *Anopheles aconitus* to malaria transmission in the central plain of Thailand. Trans R Soc Trop Med Hyg.

[19] Green CA, Rattanarithikul R, Pongparit S, Sawadwongporn P, Baimai V (1991). A newly-recognized vector of human malarial parasites in the Oriental region, *Anopheles* (Cellia) *pseudowillmori* (Theobald, 1910). Trans R Soc Trop Med Hyg.

[20] Somboon P, Aramrattana A, Lines J, Webber R (1998). Entomological and epidemiological investigations of malaria transmission in relation to population movements in forest areas of north-west Thailand. Southeast Asian J Trop Med Public Health.

[21] Thongsahuan S, Baimai V, Junkum A, Saeung A, Min GS, Joshi D (2011). Susceptibility of *Anopheles campestris*-like and *Anopheles barbirostris* species complexes to *Plasmodium falciparum* and *Plasmodium vivax* in Thailand. Mem Inst Oswaldo Cruz.

[22] Taai K, Harbach RE (2015). Systematics of the *Anopheles barbirostris* species complex (Diptera: Culicidae: Anophelinae) in Thailand. Zool J Linn Soc.

[23] Sumruayphol S, Apiwathnasorn C, Komalamisra N, Ruangsittichai J, Samung Y, Chavalitshewinkoon-Petmitr P (2010). Bionomic status of *Anopheles epiroticus* Linton & Harbach, a coastal malaria vector, in Rayong Province, Thailand. Southeast Asian J Trop Med Public Health.

[24] Takken W, Verhulst NO (2013). Host preferences of blood-feeding mosquitoes. Annu Rev Entomol.

[25] Tusting LS, Bousema T, Smith DL, Drakeley C (2014). Measuring changes in *Plasmodium falciparum* transmission: precision, accuracy and costs of metrics. Adv Parasitol.

[26] Shaukat AM, Breman JG, McKenzie FE (2010). Using the entomological inoculation rate to assess the impact of vector control on malaria parasite transmission and elimination. Malar J.

[27] Graumans W, Jacobs E, Bousema T, Sinnis P (2020). When is a *Plasmodium*-infected mosquito an infectious mosquito?. Trends Parasitol.

[28] Stevenson JC, Norris DE (2016). Implicating cryptic and novel *Anophelines* as malaria vectors in Africa. Insects.

[29] Epopa PS, Collins CM, North A, Millogo AA, Benedict MQ, Tripet F (2019). Seasonal malaria vector and transmission dynamics in Western Burkina Faso. Malar J.

[30] Stone WJ, Eldering M, van Gemert GJ, Lanke KH, Grignard L, van de Vegte-Bolmer MG (2013). The relevance and applicability of oocyst prevalence as a read-out for mosquito feeding assays. Sci Rep.

[31] Moher D, Liberati A, Tetzlaff J, Altman DG, Group P (2009). Preferred reporting items for systematic reviews and meta-analyses: the PRISMA statement. PLoS Med..

[32] von Elm E, Altman DG, Egger M, Pocock SJ, Gotzsche PC, Vandenbroucke JP (2007). The strengthening the reporting of observational studies in epidemiology (STROBE) statement: guidelines for reporting observational studies. PLoS Med.

[33] DerSimonian R, Laird N (1986). Meta-analysis in clinical trials. Control Clin Trials.

[34] Nyaga VN, Arbyn M, Aerts M (2014). Metaprop: a Stata command to perform meta-analysis of binomial data. Arch Public Health.

[35] Hunter JP, Saratzis A, Sutton AJ, Boucher RH, Sayers RD, Bown MJ (2014). In meta-analyses of proportion studies, funnel plots were found to be an inaccurate method of assessing publication bias. J Clin Epidemiol.

[36] Brusich M, Grieco J, Penney N, Tisgratog R, Ritthison W, Chareonviriyaphap T (2015). Targeting educational campaigns for prevention of malaria and dengue fever: an assessment in Thailand. Parasit Vectors.

[37] Frances SP, Klein TA, Wirtz RA, Eamsila C, Pilakasiri C, Linthicum KJ (1996). *Plasmodium falciparum* and *P. vivax* circumsporozoite proteins in anophelines (Diptera: Culicidae) collected in eastern Thailand. J Med Entomol.

[38] Gingrich JB, Weatherhead A, Sattabongkot J, Pilakasiri C, Wirtz RA (1990). Hyperendemic malaria in a Thai village: dependence of year-round transmission on focal and seasonally circumscribed mosquito (Diptera: Culicidae) habitats. J Med Entomol.

[39] Harbach RE, Gingrich JB, Pang LW (1987). Some entomological observations on malaria transmission in a remote village in Northwestern Thailand. J Eur Mosq Control Assoc.

[40] Limrat D, Rojruthai B, Apiwathnasorn C, Samung Y, Prommongkol S (2001). *Anopheles barbirostris*/*campestris* as a probable vector of malaria in Aranyaprathet, Sa Kaeo Province. Southeast Asian J Trop Med Public Health.

[41] Poolphol P, Harbach RE, Sriwichai P, Aupalee K, Sattabongkot J, Kumpitak C (2017). Natural *Plasmodium vivax* infections in *Anopheles* mosquitoes in a malaria endemic area of Northeastern Thailand. Parasitol Res.

[42] Rattaprasert P, Chaksangchaichot P, Wihokhoen B, Suparach N, Sorosjinda-Nunthawarasilp P (2016). Detection of putative antimalarial-resistant *Plasmodium vivax* in *Anopheles* vectors at Thailand-Cambodia and Thailand-Myanmar borders. Southeast Asian J Trop Med Public Health.

[43] Sattabongkot J, Kiattibut C, Kumpitak C, Ponlawat A, Ryan JR, Chan AST (2004). Evaluation of the VecTest Malaria Antigen Panel assay for the detection of *Plasmodium falciparum* and *P. vivax* circumsporozoite protein in anopheline mosquitoes in Thailand. J Med Entomol.

[44] Sriwichai P, Karl S, Samung Y, Kiattibutr K, Sirichaisinthop J, Mueller I (2017). Imported *Plasmodium falciparum* and locally transmitted *Plasmodium vivax*: cross-border malaria transmission scenario in Northwestern Thailand. Malar J.

[45] Sriwichai P, Samung Y, Sumruayphol S, Kiattibutr K, Kumpitak C, Payakkapol A (2016). Natural human *Plasmodium* infections in major *Anopheles* mosquitoes in Western Thailand. Parasit Vectors.

[46] Sumarnrote A, Corbel V, Overgaard HJ, Celhay O, Marasri N, Fustec B (2018). *Plasmodium* infections in *Anopheles* mosquitoes in Ubon Ratchathani Province, Northeastern Thailand during a malaria outbreak. J Am Mosq Control Assoc.

[47] Tainchum K, Ritthison W, Chuaycharoensuk T, Bangs MJ, Manguin S, Chareonviriyaphap T (2014). Diversity of *Anopheles* species and trophic behavior of putative malaria vectors in two malaria endemic areas of Northwestern Thailand. J Vector Ecol.

[48] Zollner G, Sattabongkot J, Vaughan JA, Kankaew P, Robert LL, Thimasarn K (2016). Longitudinal evaluation of malaria epidemiology in an isolated village in Western Thailand: I. Study site and adult anopheline bionomics. Southeast Asian J Trop Med Public Health.

[49] Baker EZ, Beier JC, Meek SR, Wirtz RA (1987). Detection and quantification of *Plasmodium falciparum* and *P. vivax* infections in Thai-Kampuchean Anopheles (Diptera: Culicidae) by enzyme-linked immunosorbent assay. J Med Entomol.

[50] Carrara VI, Sirilak S, Thonglairuam J, Rojanawatsirivet C, Proux S, Gilbos V (2006). Deployment of early diagnosis and mefloquine-artesunate treatment of falciparum malaria in Thailand: the Tak Malaria Initiative. PLoS Med.

[51] Coleman RE, Barth JF, Turell MJ, Gordon SW, Sattabongkot J, Copeland R (2000). Development and evaluation of a dipstick assay for detection of *Plasmodium falciparum* and *P. vivax* sporozoites in mosquitoes (Diptera: Culicidae). J Med Entomol.

[52] Eamkum P, Sungvornyothin S, Kritpetcharat O, Daduang J, Lek-Uthai U, Charerntanyarak L (2014). A single-round multiplex PCR assay for the identification of *Anopheles minimus* related species infected with *Plasmodium falciparum* and *Plasmodium vivax*. Parasitol Int.

[53] Edwards HM, Sriwichai P, Kirabittir K, Prachumsri J, Chavez IF, Hii J (2019). Transmission risk beyond the village: entomological and human factors contributing to residual malaria transmission in an area approaching malaria elimination on the Thailand-Myanmar border. Malar J.

[54] Junkum A, Pitasawat B, Tuetun B, Saeung A, Rattanachanpichai E, Jariyapan N, Komalamisra N, Mogi M, Chaithong U, Choochote W (2007). Seasonal abundance and biting activity of *Anopheles Aconitus* (Diptera: Culicidae) in Chiang Mai, northern Thailand. Southeast Asian J Trop Med Public Health.

[55] Nosten F, van Vugt M, Price R, Luxemburger C, Thway KL, Brockman A (2000). Effects of artesunate-mefloquine combination on incidence of *Plasmodium falciparum* malaria and mefloquine resistance in western Thailand: a prospective study. Lancet.

[56] Ratanatham S, Upatham ES, Prasittisuk C, Rojanasunan W, Theerasilp N, Tremongkol A (1988). Bionomics of *Anopheles minimus* and its role in malaria transmission in Thailand. Southeast Asian J Trop Med Public Health.

[57] Sithiprasasna R, Linthicum KJ, Liu GJ, Jones JW, Singhasivanon P (2003). Some entomological observations on temporal and spatial distribution of malaria vectors in three villages in northwestern Thailand using a geographic information system. Southeast Asian J Trop Med Public Health.

[58] Sumruayphol S, Chaiphongpachara T, Samung Y, Ruangsittichai J, Cui L, Zhong D (2020). Seasonal dynamics and molecular differentiation of three natural *Anopheles* species (Diptera: Culicidae) of the Maculatus group (Neocellia series) in malaria hotspot villages of Thailand. Parasit Vectors.

[59] Upatham ES, Prasittisuk C, Ratanatham S, Green CA, Rojanasunan W, Setakana P (1988). Bionomics of *Anopheles maculatus* complex and their role in malaria transmission in Thailand. Southeast Asian J Trop Med Public Health.

[60] Wilkinson R, Miller TA, Esah S (1970). Anthropophilic mosquitoes in central Thailand, with notes on *Anopheles balabacensis* Baisas and malaria. Mosq News.

[61] Somboon P, Rattanarithikul R (2013). Mosquito surveys, rearing, preservation of mosquito specimens and identification of *Anopheles* in Thailand 2013.

[62] Bureau of Vector-Borne Diseases (2005). Annual Report 2005.

[63] Manguin S, Garros C, Dusfour I, Harbach RE, Coosemans M (2008). Bionomics, taxonomy, and distribution of the major malaria vector taxa of *Anopheles* subgenus Cellia in Southeast Asia: an updated review. Infect Genet Evol.

[64] Warrell DA, Gilles HM (2002). Essential Malariology.

[65] Sudathip P, Kitchakarn S, Shah JA, Bisanzio D, Young F, Gopinath D (2021). A foci cohort analysis to monitor successful and persistent foci under Thailand's Malaria elimination strategy. Malar J.

[66] Hendershot AL, Esayas E, Sutcliffe AC, Irish SR, Gadisa E, Tadesse FG (2021). A comparison of PCR and ELISA methods to detect different stages of *Plasmodium vivax* in *Anopheles arabiensis*. Parasit Vectors.

[67] Lardeux F, Tejerina R, Aliaga C, Ursic-Bedoya R, Lowenberger C, Chavez T (2008). Optimization of a semi-nested multiplex PCR to identify *Plasmodium* parasites in wild-caught *Anopheles* in Bolivia, and its application to field epidemiological studies. Trans R Soc Trop Med Hyg.

[68] Beier JC, Perkins PV, Koros JK, Onyango FK, Gargan TP, Wirtz RA (1990). Malaria sporozoite detection by dissection and ELISA to assess infectivity of Afrotropical *Anopheles* (Diptera: Culicidae). J Med Entomol.

[69] Wirtz RA, Zavala F, Charoenvit Y, Campbell GH, Burkot TR, Schneider I (1987). Comparative testing of monoclonal antibodies against *Plasmodium falciparum* sporozoites for ELISA development. Bull World Health Organ.

[70] Durnez L, Van Bortel W, Denis L, Roelants P, Veracx A, Trung HD (2011). False positive circumsporozoite protein ELISA: a challenge for the estimation of the entomological inoculation rate of malaria and for vector incrimination. Malar J.

[71] Bass C, Nikou D, Blagborough AM, Vontas J, Sinden RE, Williamson MS (2008). PCR-based detection of *Plasmodium* in *Anopheles* mosquitoes: a comparison of a new high-throughput assay with existing methods. Malar J.

[72] Appawu MA, Bosompem KM, Dadzie S, McKakpo US, Anim-Baidoo I, Dykstra E (2003). Detection of malaria sporozoites by standard ELISA and VecTestTM dipstick assay in field-collected anopheline mosquitoes from a malaria endemic site in Ghana. Trop Med Int Health.

[73] Maia MF, Kapulu M, Muthui M, Wagah MG, Ferguson HM, Dowell FE (2019). Detection of *Plasmodium falciparum* infected *Anopheles gambiae* using near-infrared spectroscopy. Malar J.

[74] Cohuet A, Harris C, Robert V, Fontenille D (2010). Evolutionary forces on *Anopheles*: what makes a malaria vector?. Trends Parasitol.

[75] Zollner GE, Ponsa N, Garman GW, Poudel S, Bell JA, Sattabongkot J (2006). Population dynamics of sporogony for *Plasmodium vivax* parasites from western Thailand developing within three species of colonized *Anopheles* mosquitoes. Malar J.

[76] Norris DE, Shurtleff AC, Touré YT, Lanzaro GC (2001). Microsatellite DNA polymorphism and heterozygosity among field and laboratory populations of *Anopheles gambiae* ss (Diptera: Culicidae). J Med Entomol.

[77] Riehle MM, Markianos K, Niaré O, Xu J, Li J, Touré AM (2006). Natural malaria infection in *Anopheles gambiae* is regulated by a single genomic control region. Science.

