# Supplementary material for: Prevalence of Plasmodium spp. in Anopheles mosquitoes in Thailand: a systematic review and meta-analysis
Source: Parasit Vectors. 2022 Aug 6;15:285. doi: 10.1186/s13071-022-05397-2 (PMC9357324; doi:10.1186/s13071-022-05397-2)
Supplement: Supplementary file 1 — Additional file 1: Table S1. Search terms [file 13071_2022_5397_MOESM1_ESM.docx]

Prevalence of *Plasmodium* spp. in *Anopheles* mosquitoes in Thailand: a systematic review and meta-analysis

Chutipong Sukkanon ^1^, Frederick Ramirez Masangkay ^2^, Wanida Mala ^1^, Kwuntida Uthaisar Kotepui ^1^, Polrat Wilairatana ^3^, Theeraphap Chareonviriyaphap^4,5^, Manas Kotepui ^1*^

^1^ Medical Technology, School of Allied Health Sciences, Walailak University, Tha Sala, Nakhon Si Thammarat, Thailand; chutipong.su@wu.ac.th (C.S.), wanida.ma@wu.ac.th (W.M.), kwuntida.ut@wu.ac.th (K.U.K.)

^2^ Department of Medical Technology, Faculty of Pharmacy, University of Santo Tomas, Manila, Philippines; frederick_masangkay2002@yahoo.com (F.R.M.)

^3^ Department of Clinical Tropical Medicine, Faculty of Tropical Medicine, Mahidol University, Bangkok, Thailand; polrat.wil@mahidol.ac.th (P.W.)

^4^ Department of Entomology, Faculty of Agriculture, Kasetsart University, Bangkok, Thailand; faasthc@ku.ac.th (T.C.)

^5^ Royal Society of Thailand, Sanam Suea Pa, Dusit, Bangkok, Thailand

***** Correspondence Author: Manas Kotepui; manas.ko@wu.ac.th (M.K.)

**Table S1. Search term**

| **Databases** | **Search terms/Search strategy** | **Date** |
| --- | --- | --- |
| PubMed | (malaria OR Plasmodium) AND (anopheles OR anopheline) AND (Thailand OR Thai OR Siam)  Search option: all fields  Search results: 379 | 30 March 2021 |
| Scopus | (malaria OR Plasmodium) AND (anopheles OR anopheline) AND (Thailand OR Thai OR Siam)  Search option: Title, abstract, keywords  Search results: 286 | 30 March 2021 |
| Web of Science | (malaria OR Plasmodium) AND (anopheles OR anopheline) AND (Thailand OR Thai OR Siam)  Search option: All fields  Search results: 448 | 30 March 2021 |
